# Conductance Tunable Suspended Graphene Nanomesh by Helium Ion Beam Milling

**DOI:** 10.3390/mi11040387

**Published:** 2020-04-07

**Authors:** Fayong Liu, Zhongwang Wang, Soya Nakanao, Shinichi Ogawa, Yukinori Morita, Marek Schmidt, Mayeesha Haque, Manoharan Muruganathan, Hiroshi Mizuta

**Affiliations:** 1 School of Material Science, Japan Advanced Institute of Science and Technology, Nomi 923-1211, Japan; s1530005@jaist.ac.jp (Z.W.); s1810134@jaist.ac.jp (S.N.); marekschmidt@gmx.de (M.S.); s1620426@jaist.ac.jp (M.H.); mano@jaist.ac.jp (M.M.); 2 National Institute of Advanced Industrial Science and Technology, 1-1-1 Umezono, Tsukuba, Ibaraki 305-8560, Japan; shin1.ogawa3@gmail.com (S.O.); y.morita@aist.go.jp (Y.M.)

**Keywords:** graphene nanomesh, helium ion microscope, thermal activation energy

## Abstract

This paper demonstrates that the electrical properties of suspended graphene nanomesh (GNM) can be tuned by systematically changing the porosity with helium ion beam milling (HIBM). The porosity of the GNM is well-controlled by defining the pitch of the periodic nanopores. The defective region surrounding the individual nanopores after HIBM, which limits the minimum pitch achievable between nanopores for a certain dose, is investigated and reported. The exponential relationship between the thermal activation energy (*E*_A_) and the porosity is found in the GNM devices. Good *E*_A_ tuneability observed from the GNMs provides a new approach to the transport gap engineering beyond the conventional nanoribbon method.

## 1. Introduction

Graphene provides a unique two-dimensional (2D) electron system, which has attracted significant attention in recent years [1,2,3]. However, it has been proved that the intrinsic properties of 2D materials are highly disturbed by the surrounding materials [4,5,6]. The silicon dioxide substrate that has been commonly used in supported graphene devices could be an extrinsic source of scattering effects which therefore limit the electron mean free path in the graphene [7,8,9]. By removing the supporting part, the suspended graphene forms an ideal platform for the application of electronic and mechanical devices due to its superior mechanical stability and high carrier mobility properties [6,10]. The modern electrical circuits are built with silicon-based field-effect-transistors (FETs), which benefit from the bandgap of silicon to achieve the ON and OFF current states for logical operation [11]. Because graphene is a semi-metal without a bandgap [12,13,14], it creates obstacles in the way for graphene towards the application for field-effect-transistors (FETs). Therefore, researchers have been looking for ways to alter its electrical properties in order to achieve functionalized graphene. By micromachining the graphene into graphene nanoribbons (GNRs), an energy gap can be observed by measuring the nonlinear conductance at room temperature, which is created by the lateral confinement of the carriers [15,16,17,18]. In addition, modifying the lattice structure of graphene by irradiation to induce defects can also open the transport gap and tune the conduction of graphene [19,20,21]. However, the driving current of GNRs is too low to be a functional device in an integrated circuit [22,23]. Moreover, the defects induced by the irradiation are usually randomly distributed [20] and highly rely on the beam quality [24]. Here, the graphene nanomesh (GNM) is generated by introducing nanopores periodically on a graphene sheet, which forms a new crystalline network based on the original crystal lattice structure of the graphene [25]. It has a larger driving current and higher on–off ratio than the narrow GNR [26,27]. Most of all, GNM has been reported to observe a transport gap opening both in theoretical simulations [28,29,30] and experiments [31,32]. These results pave the way to use the GNM in electrical logic devices. In addition, GNMs have also been proposed to be a promising candidate for applications including gas sensing [33,34], phonon engineering [35,36], battery electrodes [37], and quantum technology [38]. Although these remarkable properties of GNM were previously reported, the electrical properties of the suspended GNM with systematically controlled porosity has not been clearly investigated experimentally, especially in the sub-10 nm nanopores regime. Although the GNMs can be patterned by the electron-beam irradiation, the patterning speed is relatively low, about a few seconds for single nanopore [39]. Thus, patterning the large area of GNMs via electron-beam irradiation is impractical. The focused ion beam milling overcomes the limitation of the speed and also provides a reasonable resolution to observe the nanopore array [32].

**Figure 1:**
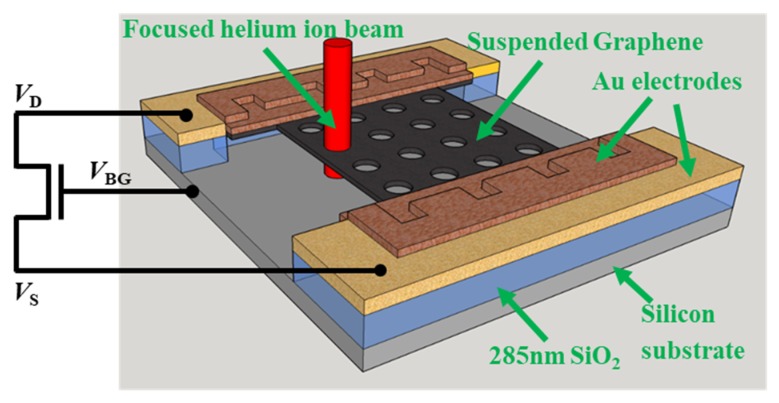
Schematic illustration of the 3D structure of the suspended GNM device. The Au electrodes on the two sides were used to apply drain voltage (*V*_D_) and source voltage (*V*_S_). The silicon substrate at the bottom was used to apply back gate voltage (*V*_BG_).

In this work, the suspended GNM devices were fabricated and patterned by focused helium ion beam milling (HIBM) (Figure 1). By optimizing the pattern location, stable suspended GNMs were obtained with different pitches of nanopores, which avoided cracking at edges during the HIBM. The electrical properties of the suspended GNM devices were measured at different temperatures. An exponential relationship was found between the porosity and the thermal activation energy of the GNMs. The results demonstrate that the GNM transport gap could be tuned by controlling the porosity.

## 2. Device Fabrication and Methods

In this section, the fabrication processes of the suspended graphene device are described. Then the structurally controlled graphene nanomesh patterning by focused helium ion beam is discussed. 

**Figure 2:**
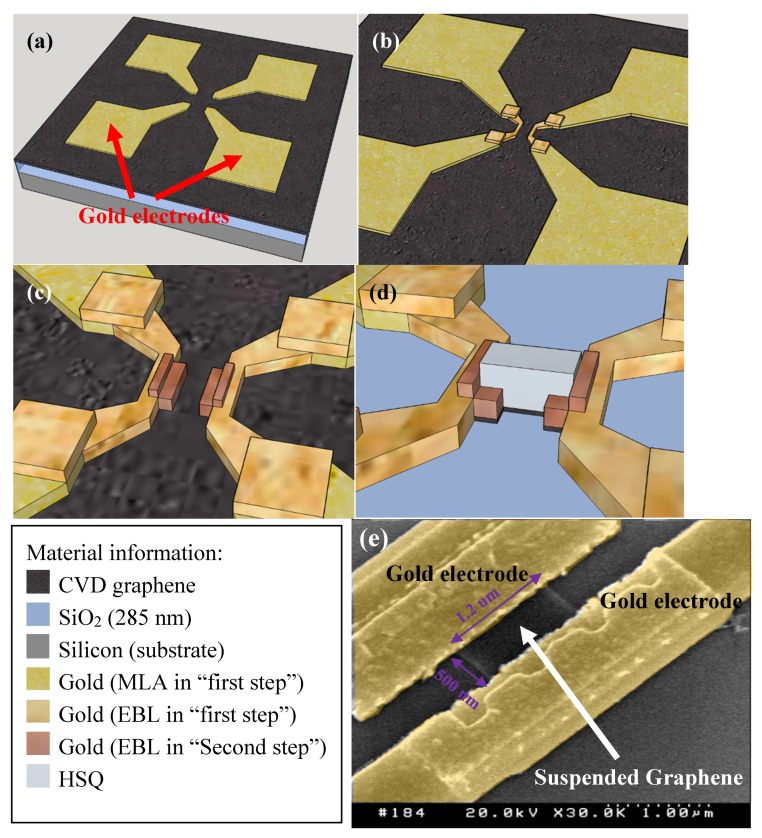
(**a**) Large gold electrodes patterning by MLA in the ‘first step’. (**b**) Small gold electrodes patterning by EBL in the ‘first step’. (**c**) The gold electrodes patterning by EBL in the ‘second step’. (**d**) The HSQ patterning by EBL and redundant graphene etching. (**e**) The SEM image of the suspended GNR device. False colored yellow regions are Au electrodes. In the middle, it remains a 500 nm long and 1.2 um wide suspended graphene.

The fabrication process started with transferring a commercial chemical vapor deposition (CVD) monolayer graphene on to a P-doped silicon substrate covered by a 285 nm thermal SiO_2_ layer. The electrodes patterning was separated into two main steps. In the first step, the aim was to pattern the electrodes with a good adhesion with the SiO_2_/Si substrate. In this case, the underneath CVD graphene was removed by O_2_ plasma etching after electrode patterning. The patterning of the electrodes was combined with a maskless aligner (MLA) and electron beam lithography (EBL) with positive photoresist. To save time, the larger electrodes pads (up to 200 um) were patterned by MLA (Figure 2a), and smaller electrodes in the center part (down to 300 nm) were patterned by EBL (Figure 2b). Then, 5 nm Cr and 80 nm Au were deposited by E-beam evaporation followed by lift-off process in acetone to form the electrodes in the first step. In the second step, the aim was to make contact between the previous electrodes layer and the CVD graphene. As the pattern sizes were as small as 500 nm, only EBL lithograph was used with positive photoresist. Then 5 nm Cr and 70 nm Au were deposited by E-beam evaporation to form the electrodes overlapping the previous electrodes and the CVD graphene (Figure 2c). Subsequently, the graphene nanoribbons (GNRs) were patterned by EBL with hydrogen silsesquioxane (HSQ). Then the CVD graphene parts which had not been covered by HSQ were removed by O_2_ plasma etching (Figure 2d). By dipping the sample into buffered hydrofluoric acid (BHF), both the HSQ over the GNRs and the SiO_2_ under the GNRs were removed. After drying the devices in a CO_2_ critical point dryer to avoid the surface tension effect, the suspended GNRs were then observed in a scanning electron microscope (SEM) (Figure 2e). The suspended GNRs were patterned in the same dimensions (length: 500 nm, width: 1.2 um) for subsequent HIBM for GNM formation. Also, some narrow suspended GNRs were patterned with different width (100 nm, 200 nm, 400 nm, 800 nm, 1200 nm) and different length (500 nm, 1 um) as the references.

**Figure 3:**
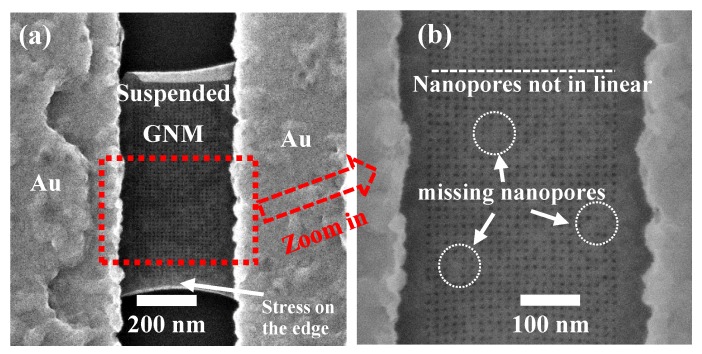
(**a**) HIB secondary electron microscopy image of typical suspended GNM devices. (**b**) 6 nm diameter nanopores and missing nanopores by zooming in at (a).

The GNMs were patterned by HIBM on the suspended GNRs in a high vacuum chamber ($<5\times 10^{-7}$ mbar), as shown in Figure 3a. In order to maintain consistent nanopore dimensions and investigate the effect of the nanopores pitch (center to center) for the electrical transport properties of the GNMs, the beam current was kept at 1 pA and the dwell time was kept at 45000 μs based on our previous work [32], where dwell time is how long the beam stays in one nanopore. After patterning, the GNMs were checked directly with the HIB secondary electron microscopy. The nanopore diameter was estimated from the magnified image contrast to be approximately 6 nm (Figure 3b). Some contaminations were found on the suspended GNMs, which prevented the helium ion beam drilling the nanopores. With this kind of large area meshing, the fraction of missing-nanopore rate was relatively low compared with our previous work [32]. In that case, the effect from missing-nanopore to the conductance of GNMs was negligible. The contaminations mainly came from the resist residue after annealing. 

**Figure 4:**
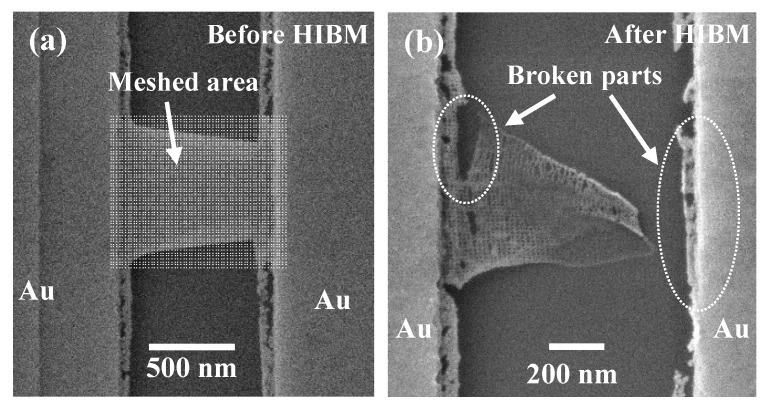
(**a**) The image shows the suspended graphene device before HIBM. The white dots square matrix is the area where the helium ion beam will drill the nanopores on, which covers all the suspended graphene area. (**b**) The image shows that the suspended graphene fell down after HIBM on the device in (a). The collapse happened in the connection part between the metal and graphene, shown as “Broken parts” in the figure.

**Figure 5:**
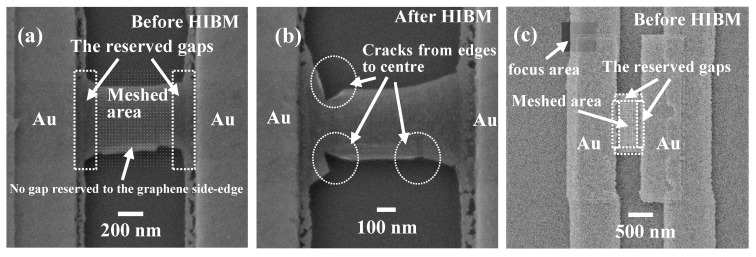
(**a**) Certain gaps reserved between the meshed area and metal layer. (**b**) The same device in (a) after HIBM, in which cracks were observed starting from the edges. (**c**) 100 nm gaps on the top and bottom, 50 nm gaps on the left and right. The focus area was located on the metal layer.

Ideally, the whole GNRs should be meshed by nanopores as shown in Figure 4a. However, due to grain boundaries and impurities [40,41,42,43], it was broken down after fully meshing on such a large area of GNR, shown as Figure 4b. It can be clearly seen that the GNMs were collapsed in the connection part between the metal and graphene (Figure 4b). In this case, a certain gap between the meshed region and the metal edges should be maintained during HIBM (Figure 5a). The result in Figure 5b showed that this kind of gap could clamp the suspended GNMs successfully. However, without any gaps remaining at the graphene side-edge (Figure 5a), the cracks formed from the edges to the center during the HIBM (Figure 5b). This was caused by the stress at the edges of the suspended graphene, which was also observed at the edges of Figure 3a. Based on our previous experience, even with the much smaller area of GNRs (both length and width were less than 300 nm), the edges of the GNRs were also found to collapse after HIBM [32]. In order to fabricate stable suspended GNMs, the meshed area for the main devices maintained a 50 nm gap to the metal edge and a 100 nm gap to the GNR side edges (Figure 5c). These 100 nm side-edge gaps, in particular, would certainly affect the conductance measurements for the meshed regions to some extent. 

**Figure 6:**
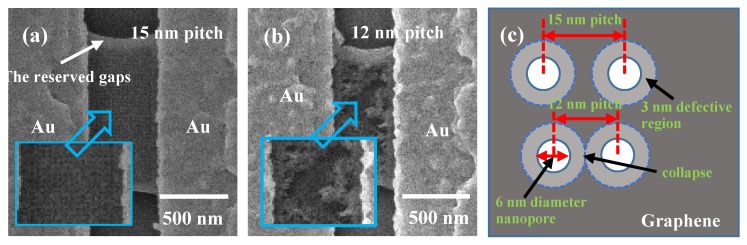
(**a**) GNM with 15 nm pitch after HIBM. (**b**) GNM with 12 pitch after HIBM. (**c**) the schematic diagram to show the defective region surrounding the nanopores. In 12 nm pitch devices, the neck almost consists of defective regions, which caused the collapse.

Porosity is an important parameter to describe the GNMs features, in particular, the phonon and electron properties [35,36,44]. By utilizing the HIBM technique, the porosity can be controlled by changing the pitch which we define as the center-to-center distance between adjacent nanopores in the meshed area. As the dose for each nanopore was fixed, the pitch was decreased from 50 nm to 30 nm, 25 nm, 20 nm, 18 nm, 15 nm, and 12 nm. It was found that the GNMs with a pitch of ≥15 nm were successfully patterned (Figure 6a). However, the GNM with a 12 nm pitch was collapsed in most of the meshed area as shown in Figure 6b. Ideally, if the nanopore diameter were still 6 nm on the 12 nm-pitch GNM device, a 6 nm-neck should remain. However, the large area collapse implied that this 6 nm-neck was highly defective and therefore unstable due to the Gaussian beam tail [24]. Under this certain dose condition, it was roughly estimated that there was a 3 nm defective region surrounding the 6 nm nanopores, which is schematically shown in Figure 6c. 

**Figure 7:**
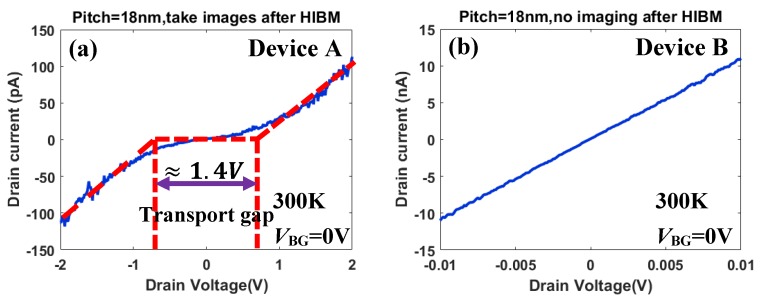
(**a**) The drain voltage (*V*_D_) dependence of the drain current (*I*_D_) with fixed back gate voltage (*V*_BG_) of 0 V at 300 K for GNM device A at 300 K. The transport gap was observed clearly due to the imaging after HIBM. (**b**) The *V*_D_ dependence of the *I*_D_ with fixed *V*_BG_ of 0 V at 300 K for GNM device B. No transport gap was observed due to no imaging after HIBM.

Another noticeable technical issue during HIBM is that the irradiation from helium ion beam imaging would introduce a certain amount of point defects on the suspended GNMs, which would affect the conductance of the GNM devices [21,45]. Two additional reference GNM devices (named Device A and Device B) were introduced, which were fabricated in the same processes with the same nanopore structure. Based on the initial electrical measurement, the GNM device A with an 18 nm pitch revealed a transport gap opening up to 1.4 V at room temperature, after imaging by the HIB secondary electron microscopy (Figure 7a). In contrast to this, another GNM device B with the same patterning (18 nm pitch) but not imaged by HIBM after patterning showed a linear feature without any obvious transport gap opening (Figure 7b) at room temperature. The structures of these two devices were identical. According to our previous results [32], the linear feature in Device B was mainly caused by the 100 nm reserved gaps in the GNR edges (Figure 6a) after HIBM. However, the imaging process by the HIB secondary electron microscopy would introduce point defects both in the 100 nm reserved gap areas and the meshed area. The conductance reduction could be orders of magnitude [21]. This is the reason why the transport gap could be observed in Device A. Unfortunately, the effect of point defects was even worse than the effect of the 100 nm gaps at the edges for investigating the transport properties of the GNMs. In this case, by ensuring an acceptable resolution for the HIBM, the smallest microscopy magnification was selected to reduce the irradiation from the HIB secondary electron microscopy. The focusing process was implemented on metal pads (Figure 5c). After HIBM, imaging was intentionally avoided to protect the GNMs devices. 

## 3. Measurement Results and Discussion

**Figure 8:**
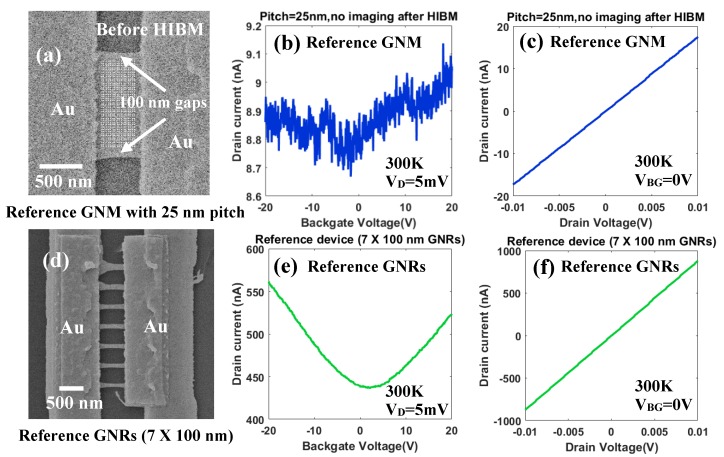
(**a**) One reference GNM device with 25 nm pitch. After HIBM, no imaging. (**b**) The *V*_BG_ dependence of the *I*_D_ with fixed *V*_D_=5 mV at 300 K for the GNM device in (a). (**c**) The *V*_D_ dependence of the *I*_D_ with fixed *V*_BG_=0 V at 300 K for the GNM device in (a). (**d**) One reference GNRs device with 7 parallel 100 nm width suspended GNRs. (**e**) The *V*_BG_ dependence of the *I*_D_ with fixed *V*_D_=5 mV at 300 K for the GNM device in (d). (**f**) The *V*_D_ dependence of the *I*_D_ with fixed *V*_BG_=0 V at 300 K for the GNM device in (d).

The GNMs were patterned with a 50 nm gap to the metal edge and a 100 nm gap to the GNR side edge, as shown in Figure 5c. No image was taken after the HIBM to protect the GNMs from the helium ions irradiation damage. The devices were measured with a cryogenic probe station in a high vacuum chamber ($<1\times 10^{-6}$ mbar). The temperature range was from 10 K to 300 K. The measurement equipment was Keithley 4200 SCS with a system noise below 1 pA. The two Au electrodes in Figure 1 were used to apply drain voltage (*V*_D_) and source voltage (*V*_S_). The chuck inside the cryogenic chamber was used to apply the back gate voltage (*V*_BG_) to the silicon substrate. The transistor performance of the GNMs devices was characterized. After HIBM, the stress on the new crystal structure of the GNM was non-uniform due to the damage in the original lattice structure. Especially, the defective region surrounding the nanopores was quite fragile. During conventional drain current versus back gate voltage ($I_{D}-V_{BG}$) measurements, a strong electrostatic force generated by *V*_BG_ was applied to the GNMs, and pulled down the suspended parts based on our previous experiments [10]. That kind of *V*_BG_ can damage the suspended GNMs and effect the conductance measurement. In this case, an additional reference GNM device was introduced to obtain the modulation of $I_{D}$ by $V_{BG}$, shown in Figure 8b. The device was 500 nm long and 1.2 um wide, shown in Figure 8a. The charge neutrality point can be extracted from the $I_{D}-V_{BG}$ measurement. To prevent the electrostatic force damaging the fragile GNMs, only conventional drain current versus drain voltage ($I_{D}-V_{D}$) measurements were implemented for all other GNM devices with the pitch from 15 nm to 50 nm. The *V*_D_ was limited to -10 mV to 10mV (Figure 8c), which avoids the joule heating effect [46]. The *V*_BG_ was set to 0 V, which is close to the charge neutrality point (Figure 8b).

**Figure 9:**
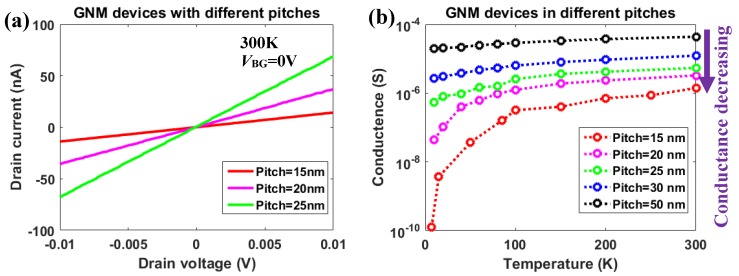
(**a**) The $I_{D}-V_{D}$ characteristics of the GNM devices with different pitches. (**b**) The conductance results from the linear fitting for the GNM devices with different pitches from 15 nm to 50 nm. The measurement temperature was from 10 K to 300 K.

It should be noted that there are 100 nm gaps on both sides of the GNM device as shown in Figure 8a. The effect of these two GNR gaps in the electrical measurement should are described below. A reference device with seven suspended GNRs (Length: 500 nm, width: 100 nm, Figure 8d) was introduced. By comparing the measurement results between the suspended GNM device and the suspended GNRs, it was observed that the back gate modulation was suppressed in the GNM device. In the Figure 8e, the average current on each 100 nm-width GNR was approximately 60 nA at *V*_BG_=0 V. However, the total current of the GNM device with two 100 nm gaps was less than 10 nA at *V*_BG_=0 V. Although there are two 100 nm gaps on both side of the GNM (Figure 8a), the total current of the GNM device was still much lower than what was expected when compared to the reference GNRs. This is thought to be caused by the strong tensile stress on the 100 nm gap region, which highly suppressed the conductance on the 100 nm gap regions [47,48,49]. After the HIBM, the original crystal structure was modified. The central meshed area released stress to the side area and sink slightly, which was confirmed by the non-linear distribution of nanopores in Figure 3b. Moreover, the 100 nm gap region was needed to clamp the whole suspended GNM device from the two sides. As a consequence of these two reasons, the highly stressed region was able to observe in the HIB secondary electron microscopy as shown in Figure 3a. As no obvious transport gap opening was observed in the GNM device (Figure 8c) comparing to our previous results [32], it implied that the stress on the 100 nm gap region was not large enough to open the bandgap and the current could still pass at room temperature. By comparing the $I_{D}-V_{D}$ measurement results of the GNM devices with different pitches (Figure 9a), the effect of changing pitches of the meshed area could still be measurable even with the 100 nm-gap regions. This proves that the two 100 nm-gap regions on the edges can be counted out of the conductance analysis for the meshed area to some extent. This also proves that the 100 nm-gap region is a notable part of the whole GNM device both in electrical properties and mechanical properties. 

**Figure 10:**
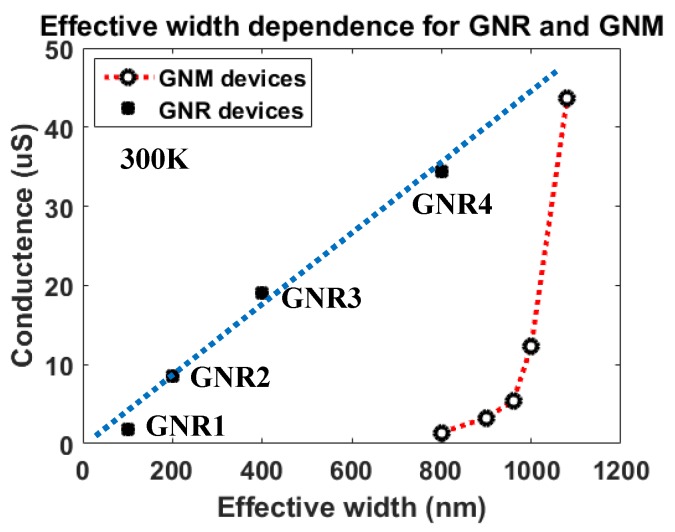
Effective width dependence for the conductance of GNR and GNM devices at 300K. The blue dotted line is the linear fitting for the GNR devices. GNR1, GNR2, GNR3, and GNR4 were the suspended GNRs with 100/200/400/800 nm width and 500 nm length.

The $I_{D}-V_{D}$ characteristics for the GNM devices exhibited a linear relationship between *V*_D_ and *I*_D_ from 20 K to 300 K, which implied that conductance was constant at a certain temperature in the limited drain voltage range. At 10K, as the temperature was too low, the conductance started increasing at higher *V*_D_ due to the Joule heating effect. In this case, the conductance of the GNM devices with different pitches at the set temperature could be extracted from the linear region. The results are shown in Figure 9b. By comparing different GNM devices at the same temperature, conductance reduction was observed. As the nanopores decrease the effective width which was defined as the sum of neck length between the adjacent nanopores, the values of the effective width were calculated for the GNM devices with various pitches and plotted in Figure 10. In contrast with the reference GNRs (GNR1: 100 nm width, 500 nm length; GNR2: 200 nm width, 500 nm length; GNR3: 400 nm width, 500 nm length; GNR4: 800 nm width, 500 nm length), the conductance of GNM devices was not in a linear relationship with the effective width. When the effective width decreased by shortening the pitches of nanopores, the conductance decreased exponentially. On the other hand, by comparing a certain GNM device at different temperatures, the conductance reduction at decreasing temperatures was much larger in the GNM devices with the smaller pitches in Figure 9b.

**Figure 11:**
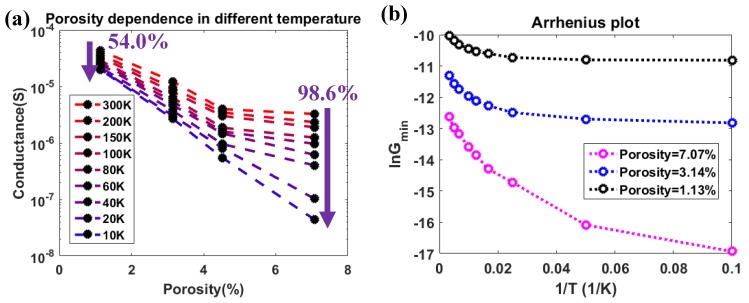
(**a**) Conductance variation of the GNM devices with different porosity from 300 K to 10 K. (**b**) Arrhenius plot example for the GNM devices with different porosities.

To investigate the heavy conductance reduction of the GNM devices, the only variable ‘nanopore pitch’ was converted to the ‘porosity’, which describes the global feature of the GNM device, shown in Figure 11a. The porosity was defined as the percentage of the nanopore areas to the total meshed area (length: 400 nm; width: 1 um). By increasing the porosity, a reduction in conductance was observed. This also clearly shows that conductance reduction by decreasing the temperature was much larger in the higher porosity devices. By decreasing the temperature from 300 K to 10 K, the conductance of GNM device with a 1.13% porosity decreased by 54.0% and that with a 7.07% porosity decreased by 98.6%. 

**Figure 12:**
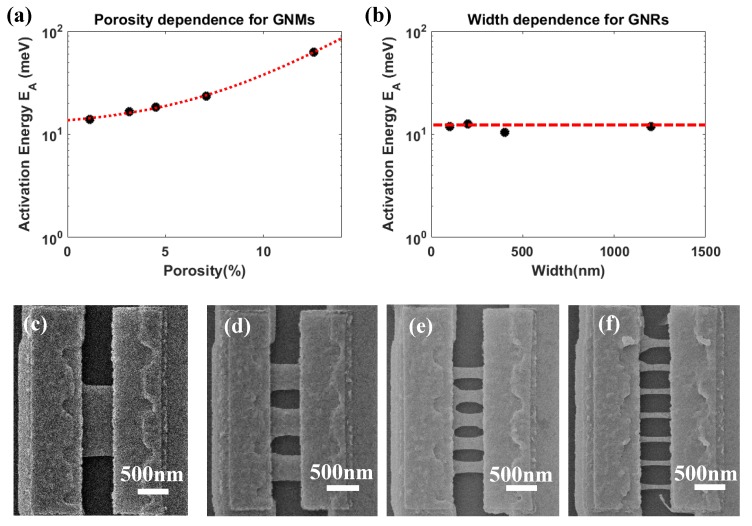
(**a**) The extracted activation energies for the GNM devices with different porosities. The black dots were the experimental data. The red dot line was the exponential fitting. (**b**) The extracted activation energies for the GNR devices with different width. The black dots were the experimental data. The red dot line was the linear fitting. (**c**) One GNR with 1200 nm width and 500 nm length. (**d**) Three GNRs with 400 nm width and 500 nm length. (**e**) Five GNRs with 200 nm width and 500 nm length. (**f**) Seven GNRs with 100 nm width and 500 nm length.

In order to investigate the nature of this conductance variation, an Arrhenius plot was used to extract the thermal activation energy. As the back gate was set around the charge neutrality point in the $I_{D}-V_{BG}$ characterization, and the conductance was almost constant in the limited $I_{D}-V_{D}$ characterization, so that the conductance extracted from the linear fitting in Figure 9a can be considered as the conductance minima ($G_{min}$) at each temperature. In each GNM device, the $G_{min}$ could be fitted to the thermal activated transport model at high temperature as

(1) $$G_{min}\propto \exp\left(-\frac{E_{A}}{2k_{B}T}\right)$$

where $E_{A}$ is the activation energy which is supposed to be the transport gap, and $k_{B}$ is the Boltzmann’s constant. The temperature dependence of $\ln G_{min}$ for a particular GNM device was shown in an Arrhenius plot in Figure 11b. By applying a linear fitting in the high-temperature regime, the $E_{A}$ values were extracted according to Equation (1). The results for the GNM devices with different porosities were plotted in Figure 12a. By fitting the experimental results, $E_{A}$ showed an exponential relationship with porosity in the GNM devices given by the phenomenological equation

(2) $$E_{A}=\alpha \exp\left(\beta *P\right)+\gamma$$

where P is the porosity of the GNM device. α, β, γ are the fitting parameters, which α = 1.918 meV; β = 0.261; γ = 11.75 meV. With the same method, the $E_{A}$ of the reference GNRs ($E_{0}$) with different values of width (larger than 100 nm) are also extracted in Figure 12b, and the devices are shown in Figure 12c–f. The $E_{0}$ is shown as a constant of 11.61 meV, which is very close to α+γ obtained above and also consistent with the reported value [50]. It proves that if the porosity were approaching zero, the exponential fitting results would be consistent with the $E_{0}$, shown as

(3) $$E_{A}\left(P=0\%\right)=\alpha +\gamma \approx \;E_{0}$$

It clearly demonstrates that $E_{A}$ increased exponentially by increasing the porosity of the GNM device. From this point of view, the conductance of the GNMs can be systematically tuned by the well-controlled HIBM meshing. The tuneability of $E_{A}$ via the porosity may be caused by quantum confinement and strong localization [21,50]. The GNMs can also be considered as small GNR array. The small GNRs in this work have already entered the sub-10 nm regime, including the defective region. The quantum confinement and edge disorder have been proved to generate the energy gaps in the small GNRs in the calculation results [51,52]. Besides, the defective region will enhance the disorder on the edges, which also contributes to the transport gap opening [15]. In this case, the tunable $E_{A}$ of the GNMs is considered to be the macroscopic expression of the quantum and localization effects of each small GNR. The similar tuneability of $E_{A}$ can be only observed in the GNRs with a size of less than 50 nm [50]. As the fabrication of ultra-scaled suspended GNRs is quite challenging, the GNMs also provide a new way to investigate the scaled suspended GNRs in the limited fabrication technique. On the other hand, the electron transport properties is dominated by variable range hopping in this kind of functionalized graphene device at low temperature [21,50]. The division in the low-temperature region from the linear fitting of the high-temperature region in the Arrhenius plot (Figure 11b) was also observed. As the necks between the nanopores have entered sub-10 nm regime, the mechanism at low-temperature will also be an interesting point for future study.

## 4. Conclusions

The electrical properties of the suspended GNM devices with various porosities were investigated experimentally. The stable suspended GNM devices were successfully fabricated with well-controlled porosities by using the HIBM technique. The defective region surrounding the nanopores caused by the Gaussian beam tail was reported, which limited the minimum pitches between the nanopores’ center. With the help of Arrhenius plot, the effect of transport opening in the GNMs was characterized, and the thermal activation energy and the porosity were observed in a nearly perfect exponential relationship. In this case, the conductance of the suspended GNM device was able to be tuned by systematically changing the porosity of the GNM due to the quantum confinement and strong localization. This leads a novel approach for GNM engineering towards applications in sensing, phonon engineering, and quantum technologies.
